# Co-designing and evaluation of a context-appropriate strategy to improve access to early detection and care of oral, breast and cervical cancers in rural India: a formative phase implementation research protocol

**DOI:** 10.3332/ecancer.2025.1849

**Published:** 2025-02-13

**Authors:** Arunah Chandran, Ishu Kataria, Kunal Oswal, Rita Isaac, Meritxell Mallafré-Larrosa, Sathishrajaa Palaniraja, Rajaraman Swaminathan, Rohit Rebello, Nandimandalam Venkata Vani, Bindhya Vijayan, Moni Kuriakose, Arnie Purushotham, Rengaswamy Sankaranarayanan, Jerard Selvam, Richard Sullivan, Partha Basu

**Affiliations:** 1 International Agency for Research on Cancer, Lyon 69366, France; 2 Center for Global Noncommunicable Diseases, RTI International, New Delhi 100037, India; 3 Karkinos Healthcare, Bengaluru 560103, India; 4 Faculty of Medicine and Health Sciences, University of Barcelona, Barcelona 08007, Spain; 5 Mailman School of Public Health, University of Columbia, New York, NY 10032, USA; 6 Cancer Institute (WIA), Chennai 600020, India; 7 Department of Medical Oncology, GBH Group of Hospital, Udaipur, Rajasthan 313001, India; 8 King’s College London, London WC2R 2LS, UK; 9 National Health Mission Tamil Nadu, Chennai 600006, India

**Keywords:** cancer, early detection, implementation research, effectiveness–implementation hybrid design, India

## Abstract

**Background:**

In India, cancers of oral cavity, breast and cervix account for more than one-third of all cancers among the Indian population and cause nearly 0.25 million deaths every year in the country. Cancer has a catastrophic impact on the rural men and women of India, the majority of whom are socioeconomically disadvantaged with one-fifth living below the national poverty line. The cancer early detection strategies adopted in India today remain suboptimal. The Access Cancer Care India (ACCI) project aims to design and evaluate a new multilevel strategy, integrated and contextualised to the local health system, to improve access to the early detection and care continuum for oral, breast and cervical cancers among the rural population in India.

**Methods:**

We propose to conduct an effectiveness–implementation hybrid research study in three distinct states of India, focusing on the rural population residing in each state. The study’s objectives will be addressed through a series of interrelated and sequential six work packages that encompass stakeholder and policy analysis, a mixed-method study to evaluate barriers and facilitators in accessing early detection services, a health system capacity assessment, a pilot implementation and evaluation and, finally, a determination of the readiness to sustain and scale the strategy. A pragmatic effectiveness–implementation hybrid design will be employed. The study has been granted regulatory clearance by the Health Ministry Screening Committee of India and obtained ethical approval from all collaborating institutions.

**Discussion:**

The ACCI project aims to establish a feasible early detection strategy for breast, cervical and oral cancers, recognizing that successful implementation and sustainability depend on stakeholder engagement, contextual analysis, capacity assessment and readiness for change. The proposed pragmatic study design addresses the challenges faced by policymakers and program managers in making evidence-based decisions. The insights gained from this study will be invaluable for other LMICs that share similar resource constraints and healthcare infrastructure challenges.

**Clinical Trial Registry India:**

CTRI/2022/09/045927.

## Background

Globally, cancers of oral cavity, breast and cervix account for approximately 15% of all cancer incidence. In India, these cancers account for one-third of all cancers and cause nearly 0.25 million deaths every year in the country [1]. The latest study that evaluated the cancer trends across the available 28 population-based cancer registries and 58 hospital-based cancer registries in India showed an increasing trend in cancer incidence across all sites in both sexes during 2012–2016. India’s first population-based rural cancer registry in Barshi, Maharashtra, also reported age-adjusted incidence rates between 50.6 per 100,000 and 61 per 100,000 among men and women in the rural population [2]. Cancer has a catastrophic impact on the rural communities in India, the majority of whom are socioeconomically disadvantaged, with one in ten living below the national poverty line [3]. The cost of treating cancer in rural India is among the highest for any disease due to the advanced stage of presentation [4]. Treatment for advanced and metastatic cancer is significantly more expensive than early stage. Treatment is primarily financed through out-of-pocket expenses, including borrowing, selling assets and financial assistance from friends and relatives [5]. Overall, 60% of households seeking care in the private sector experience catastrophic expenses. Fatality rates are also significantly higher for cancers in rural populations than in urban [6]. Access to cancer early detection and subsequent care is limited in rural India, due to geographical, infrastructural and financial barriers. While 70% of cancer patients live in rural areas, 95% of cancer care facilities are concentrated in urban centres, forcing patients to travel long distances for diagnosis and treatment. In addition, the rural population in India faces several sociocultural barriers such as stigma and fear of cancer. Coupled with the financial burden and limited health infrastructure in rural health centres, these urban–rural disparities have contributed to increased treatment dropout rates among the rural population [7].

The cancer early detection strategies adopted in the national screening program in India and its subsequent implementation are suboptimal [8]. Despite compelling evidence on targeted screening of users of tobacco or alcohol or both, guidelines recommend oral cancer screening for all. The Indian recommendation for screening with clinical breast examination (CBE) every 5 years is also not evidence-based as both the randomized controlled trials (RCTs) evaluating CBE as a screening test in India have demonstrated benefits to achieve downstaging and improved survival when screening is done every 2–3 years [9, 10]. Starting screening at the age of 30 years for breast cancer is likely to cause more harm than benefits. Visual inspection of cervix after acetic acid application has been recommended as a screening test for cervical cancer in the Indian guidelines following the significant reduction (exceeding 30%) in mortality demonstrated by two RCTs conducted in the country [11, 12]. However, translating VIA from research to real-life programmes has been challenging due to the subjective nature of the test and the difficulties in ensuring quality. The National Family Health Survey (NHFS. 2023) reveals the woefully low coverage of screening (<2% for any cancer site) [13]. Improving the implementation of cancer early detection services in public health settings coupled with addressing downstream management pathways is key to improving the scenario in India. Oral cancer screening needs to target high-risk users above 35 years of age as effectiveness and cost-effectiveness have been demonstrated in this subpopulation only [14–16]. For breast cancers, the WHO recommends to prioritise an ‘early diagnosis’ approach (reducing delays in diagnosis and treatment of symptomatic individuals) over screening [17], while for cervical cancer, the WHO recommends HPV detection-based screening followed by treatment as part of elimination strategy. The early diagnosis approach is relevant to improve survival for oral and cervical cancer as well [18].

Introduction of the new interventions requires innovative implementation strategies to create awareness, improve uptake and referral rates and reduce delays in the early detection pathway. Implementation research that can identify the barriers faced by the health providers, organisations and target population in the early detection of cancers can help formulate feasible strategies to improve programmes. There are several evidence-based strategies that are applicable in the current setting, such as self-collection of samples for HPV detection or the use of trained community health workers (CHWs) to create awareness about the importance of early detection of cancers or to perform oral cancer screening [19–22]. However, designing the implementation strategies for such evidence-based interventions requires a thorough understanding of the target population, the structural barriers they encounter and the local health systems. Involving local stakeholders is the key to design context-appropriate and sustainable solutions. The Access Cancer Care India (ACCI) project aims to improve early cancer diagnosis and screening through stakeholder engagement, barrier identification and capacity building.

### Objective

In this implementation research study, we aim to design and evaluate a new multilevel strategy to improve access to the early detection and care continuum for oral, breast and cervical cancer among the rural population in India that is integrated and contextualised to the local health system. In the pre-intervention phase, we aim: 1) to determine the capacity of the current health services for early detection of oral, breast and cervical cancer among rural population in India and 2) to determine barriers and facilitators for access to early detection and treatment services for oral, breast and cervical cancer among rural population in India.

Subsequently, in the design and evaluation phases, we aim: 1) to co-design and cost a multilevel intervention strategy to improve early detection (screening and early diagnosis) of oral, breast and cervical cancer among rural population in India in consultation with stakeholders and 2) to pilot the intervention strategy and evaluate the same for implementation outcomes such as adoption, fidelity and sustainability and also assess their effectiveness to improve early detection of oral, breast and cervical cancer among rural population in India.

## Methodology

### Study setting

We propose to conduct our study in three different states in India, targeting the rural population. The study sites were purposefully chosen to represent a range of population characteristics, cancer burdens and health system structures and organization.

#### Kerala

This southern state of India has the lowest proportion of multidimensional poor population at 0.71% compared to the national average of 25.01% [23]. Based on the national Sustainable Development Goal (SDG) Index survey, Kerala ranked the highest in the country, with a score of 70 [18]. The health services in this state are better organized compared to other states in India. While ten of the fourteen districts in Kerala report less than a 10% poverty rate, the selected Idukki district is one of the districts with higher poverty rates (population according to Census 2011: 1,056,929, 95% rural). It is the second largest district covering an area of 4,358 sq.km, constituting 11.2% of the total area of the state. The district is covered with dense tropical forests, plantations, scrublands and grasslands with most of the population income derived from agriculture and poor rail routes. Administratively, the district is divided into five taluks (Devikulam, Peerumedu, Udumbanchola, Thodupuzha and Idukki). There are co-existing public and private healthcare facilities with no major cancer care institutions. Breast, cervix and oral cancer are the first, second and fifth common cancers in Kerala, respectively (crude disability-adjusted life year (DALY) rates: 240.8 (breast), 82·3 (cervix) and 171 (oral) per 100,000 in 2016). The state of Kerala had sporadic cancer screening and early detection campaigns arranged by the participating institution in specific sites [24, 25].

#### Tamil Nadu

This southern state of India has a proportion of 4.89% of multidimensional poor, ranking 4th lowest [17]. The state has a well-organized health system and ranks third on the national SDG Index with a score of 67. It also has the longest running cancer screening program in India. Crude incidence and age-standardized cancer incidence rates in Tamil Nadu are 96.1 and 86.4 per 100,000, respectively. This present study is planned at the Viluppuram district (population according to Census 2011: 3,458,873) of Tamil Nadu, which has a predominantly rural population (86%) and a high cancer burden. It has nine taluks (Viluppuram, Vikkiravandi, Vanur, Kandachipuram, Thiruvennainallur, Tindivanam, Gingee, Melmalaiyanur and Marakkanam). The Tamil Nadu Cancer Registry Project in 2016 reported cervical cancer followed by breast cancer to be the most common cancer among women in the Viluppuram district. Oral cancer was the third most common cancer among both males and females. The population coverage of the state-run screening program is low and an organized screening program undertaken by the Cancer Institute (WIA) is in progress. Tamil Nadu has an ongoing government-run facility-based opportunistic cancer screening program for cervical and breast cancer since 2012 and an oral cancer screening program since 2017. Apart from this, an organized community-based cancer screening program has been conducted by the participating institution since 2015 [24, 25].

#### Rajasthan

This northwestern Indian state has a large area covered by desert with many impoverished rural communities. It has a proportion of 29.46% of multidimensional poor and ranks in the bottom half among the Indian states, with an SDG Index Survey Score of 57 [23, 26]. The present study will be conducted in two neighbouring Udaipur (population according to Census 2011: 3,068,420 and rural: 80%) and Rajsamand districts (population according to Census 2011: 1,156,597 and rural: 84%) of Rajasthan [24]. There are co-existing public and private healthcare facilities in these districts. Udaipur and Rajsamand are divided into 20 and 8 taluks, respectively. Breast and cervical cancer are the first and third most common cancers in Rajasthan (crude DALY rates: 124 (breast), 83.9 (cervix) and 100 (oral) per 100,000 in 2016). The state of Rajasthan did not have any cancer screening activity [25].

### Study design

**Figure 1.**
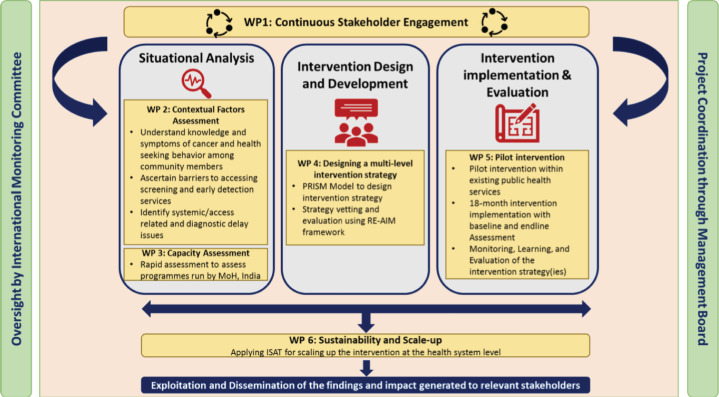
Flow chart of interactions of Work Packages.

The objectives of this study will be addressed through a series of interconnected and sequential work packages (WPs) as outlined in Figure 1, conducted across two phases. The pre-intervention phase (WP 1-3) constitutes stakeholder engagement, identifying contextual factors, assessing barriers and facilitators and health system capacity assessment focusing primarily on improving cancer early detection services. The piloting phase (WP 4-6) constitutes co-designing, implementation and evaluation WPs. The information obtained in the pre-intervention phase will be utilised to co-design the strategies for implementation with stakeholders. After implementation, during WP5 and WP6 of this phase, we will apply the Practical, Robust Implementation and Sustainability Model (PRISM) framework to assess multilevel contextual factors and reach, effectiveness, adoption, implementation and maintenance (RE-AIM) framework for the planning and evaluation of outcomes of the programme [27, 28]. The detailed study timeline is mentioned in Supplementary file 1.

### Pre-intervention phase

#### WP1: stakeholder analysis and engagement

Early detection programmes for cancer are complex interventions involving a wide range of stakeholders; at the macro (policymakers, funders, donors, programme coordinators, administrators of district hospital and oncology centres), meso (service providers, civil society organizations, special interest groups and CHW) and micro levels (community members, oral, breast and cervical cancer patients). A stakeholder matrix tool will be adapted and applied by the research team to create site-specific stakeholder groups [29].

Each of the stakeholders’ roles, influence and interests in the selected strategy, related programmes and policies will be defined. The stakeholders will be classified according to how they can be affected by actions and how they can affect the actions. They will be formally engaged during the planning, implementation and evaluation process. This analysis will be a living document that will be updated by the research team with stakeholders that may not have been initially considered as the project progresses. These stakeholders will be consulted in workshops, meetings and informal group discussions. Power relations in group interactions will be addressed carefully in settings where some participants have prior experience in engaging in academic policy and practice discussions and others do not. A tool for stakeholder engagement will be developed to collect, collate and analyse the inputs from the stakeholders in planning, implementing and evaluating the intervention strategies.

#### WP2: assessing barriers and facilitators in implementing a CHW-led early detection strategy and treatment services for oral, breast and cervical cancers

This WP focuses on understanding the context-specific barriers, challenges and opportunities that exist for rural men and women to access the cancer early detection care continuum through a mixed-method approach. This WP encompasses the entire pathway of early detection – community mobilization for screening, access to screening and diagnostic services and navigating the systems to initiate treatment. The contextual factors at individual, provider and systems levels will be examined using the rapid assessment and response evaluation (RARE) methodology, which is a rapid ethnographic assessment to elicit beliefs and perceptions around health prevention and treatment, including assessing barriers [30]. The following components of the RARE technique will be used to study the knowledge, attitude, perceptions and beliefs of the target population: a) key informant interview (KII); b) focus group discussions (FGDs) and c) questionnaire survey. At each study site, we will identify blocks where the pilot intervention will be implemented (discussed later). The contextual factors and barrier assessment study will be implemented in these blocks.

The assessment will involve the following tasks at each study site.

- Questionnaire surveys will be among two groups of participants. The first survey will be conducted among men and women aged 30–65 years to understand their knowledge and understanding of common symptoms of cancer, health-seeking behaviour, their choices related to screening and diagnostic services, myths and beliefs, access to the health facilities, perceptions of staff attitude, expenses on diagnosis, treatment and so on. The second survey will be administered to breast, cervical and oral cancer patients in the tertiary care centres. Using a structured questionnaire, their journey from disease onset to the final diagnosis will be documented to estimate the access and diagnostic delays as well as the possible reasons for such delays. The research assistants at each site will be responsible for administering the survey questionnaires. All surveys are interviewer-administered using paper-based forms on a one-to-one basis and the data will be transferred into the electronic REDCap database.
- FGDs will be conducted among both the general population and CHWs, at each site with a group size not exceeding 6–8 persons. CHWs, including Accredited Social Health Activists, Village Health Nurses and Auxiliary Nurse Midwives, will be chosen. They play a crucial role in addressing healthcare gaps in rural India and serve as vital connections between the community and the health system, acting as primary points of contact for health promotion initiatives and patient engagement [31]. The FGDs will explore barriers to accessing early detection services and factors affecting referral uptake. Views on potential solutions/interventions will be explored. The participants for the general population FGDs will be selected purposively by the local community leaders. FGDs with CHWs will explore participant knowledge and understanding of common cancer symptoms, screening guidelines and referral practices. The current roles and responsibilities, working patterns, challenges they perceive to provide home-based screening services, training needs and capacity to navigate patients will also be explored. The participants for the CHWs’ FGDs will be identified in consultation with the local stakeholder team.
- KIIs will be held with the head of the panchayat (rural administrative body), chief medical officer, state cancer coordinator and the administrators of the local tertiary care facilities. Through these interviews, the status of the implementation of the cancer screening programme, the barriers for the rural men and women to participate, the future plans for improvement and the challenges of modifying the existing system to introduce new concepts such as CHW-driven early detection strategy will be explored.

#### WP3: capacity assessment of the local health systems in implementing a CHW-led early detection strategy for oral, breast and cervical cancers

It is vital to assess the organizational readiness for the intervention and proposed implementation strategy, focusing on whether it addresses the barriers of the stakeholders, the need for coordination across the cancer care continuum, complexity and resources required and the usability of the strategy. The PRISM model will be used to guide organisational assessment for implementation [32] using in-depth interviews with key informants. Further in-depth capacity assessment will also be performed in the selected study sites. We will adapt the CervScreen-SARA tool developed by the International Agency for Research on Cancer (IARC) based on the WHO-SARA assessment as these are tailored to assess programmes run nationally and regionally and are participatory in nature [33]. In short, we will perform the capacity assessment according to the following steps.

- Engage the relevant stakeholders and plan the health system capacity assessment jointly.
- List the scope of the capacities to be assessed across the building blocks of the health system.
- Conduct a desk review to examine the current national, state and district cancer policies from multiple sources in preparation for stakeholder engagement: literature databases, search engines, targeted websites and consultations with content experts for potentially relevant documents (reports, guidelines, policy and program documents).
- Perform site visits to selected service delivery points to collect facility readiness information using a structured questionnaire.
- Review and confirm findings with the stakeholders through KII to compare the desired capacities against existing capacities and resources to determine the level of effort required to bridge the gap between them.

### Piloting phase

#### WP4: co-designing and costing a multilevel intervention strategy to improve participation of the rural populations in the early detection care continuum

We will use the PRISM model to design a sustainable and contextualized implementation package comprising multilevel strategies and evaluate the same using the RE-AIM framework. The model considers how the external environment, intervention design, implementation infrastructure and adopting organization (with special emphasis on the health care providers) and its patients influence the implementation and success of the strategies. Furthermore, we will conduct an ingredient-based costing of the implementation strategies that will inform district-level budget planning.

The findings of WP1–WP3 will be synthesized and triangulated to feed the ‘intervention’ domain of the PRISM and will be presented to the stakeholders’ team workshop. In collaboration with the stakeholders, we will identify the intervention elements and strategies that are ‘best fit’ from the perspectives of the organization and populations/patients to be targeted.

The interventions and strategies may be different to suit the local context in the three target districts and will be selected from a ‘menu’ of options. The ‘menu’ will incorporate several evidence-based options that have been trialled elsewhere, such as a CHW-driven home-based early detection and navigation approach, HPV detection-based screening relying predominantly on self-sampling, shifting from systematic CBE screening to an ‘early diagnosis’ approach and training of frontline health providers (CHWs, nurses, clinicians at primary and secondary levels) in detecting the ‘warning’ symptoms of common cancers. We also propose to develop a setting-appropriate referral guideline from primary care to secondary care and finally to oncology centres utilizing the online portal developed by the National Cancer Grid India [34] for training, navigating the men and women who are screen-positive and/or have ‘warning’ symptoms through diagnostic and treatment pathways and are likely to be included in the ‘menu’. The navigation strategy may be selected from various options such as navigation by CHWs or trained social workers, assignment of designated nurse at health facilities, telephone helplines run by the cancer survivors, trained counsellors or social workers, creation of community volunteer groups or provision of transport services.

Once the strategies that are to be incorporated in the implementation package are identified, we will develop the protocol, the logic model and other toolkits that will be necessary for piloting and evaluating the package.

This WP will include a costing toolkit that allows for district-level ingredient-based costing of the strategies. The toolkit will be modelled after the Global HEARTS costing tool [35]. Costs include human resources and requisite training, equipment and supplies, the depreciated value of structures and vehicles and a cost estimate of the contribution of other health system components. Costs will be collected during the rollout of the pilot, and then, the cost expansion path will be estimated based on the scale-up scenario described in WP6. Indicators of affordability at the district and state levels will be provided based on the variables that determine health budgets for the relevant jurisdictions. These variables may include catchment population, disease burden in the population, a number of health facilities, level of infrastructure at health facilities and human resource capacity.

#### WP5: pilot implementation and evaluation of implementation package

The implementation strategy for the interventions will be co-designed with the stakeholders and nested into the existing public health services in each selected district. We will implement the strategies through a single-arm trial in four randomly chosen blocks in each district. Data will be captured during pre-intervention, intervention and post-intervention period. Data collected from the initiation of interventions will show whether any baseline secular trends have changed in the blocks (before and after intervention).

In addition, the costing protocol will be tested during pilot implementation of the intervention strategies and adapted as needed to the existing public health infrastructure. The adaptation will produce a custom costing tool for on-going use in each district that will support local officials for budgeting purposes.

We will define and measure outputs (direct products of activities and will include types, levels and targets of services to be delivered) and SMART outcome indicators such as specific changes in health-seeking behaviour, changes in health providers’ knowledge and skills, adoption of acquired skills, access and participation to cancer early detection and treatment and diagnostic intervals in the selected blocks. Similar indicators measured in the pre-intervention period will allow us to correlate between the interventions and their impact. Most of these outcomes will be implementation outcomes, though we will measure effectiveness outcomes such as interval between symptom onset and diagnosis confirmation or interval between screening and further assessment/treatment, detection and stage of precancers and cancers and so on.

#### WP6: assessment of setting’s readiness and capacity to sustain and scale up interventions

We will use the validated Intervention Scalability Assessment Tool (ISAT), which is designed to assist practitioners, policymakers, programme managers and researchers to determine the scalability of a discrete health programme or intervention [33]. The tool consists of three sections and each section has multiple domains.

- Part A: It consists of five domains: 1) the problem; 2) the intervention; 3) strategic and political context; 4) evidence of effectiveness and 5) intervention costs and benefits.
- Part B: It consists of five domains: 1) fidelity and adaptation; 2) reach and acceptability; 3) delivery setting and workforce; 4) implementation infrastructure and 5) sustainability.
- Part C: It summarizes all the information gathered to facilitate the process of making a recommendation on scalability.

### Sampling methodology and sample size estimation

WP2 involves a population survey. A multistage cluster sampling design will be employed to select participants for the surveys. Blocks will serve as the primary sampling units and households as the secondary sampling units. Based on an expected 50% prevalence of knowledge and understanding about common cancer symptoms and assuming a 10% precision, 95% confidence level and 20% nonresponse rate, a total sample size of 480 is estimated for each state [36]. This sample size will be proportionally divided among randomly selected blocks. Subsequently, the number of households from each block will be proportionately distributed using systematic sampling from a list of households. One respondent from each household, alternating male and female, will be chosen to get equal male and female participants. The number of participants required for the hospital-based survey in WP2 has been empirically decided at 100 patients with breast, cervical and oral cancer from each study site. It is estimated that a minimum of six FGDs per state will be conducted or more until saturation is achieved.

For WP3, the services will be broadly categorised into primary, secondary and tertiary healthcare settings. The number of facilities to be sampled will be calculated using the standard formula for a proportion using an expected percentage of health facilities offering a given service as the outcome. An expected proportion of 90% of service availability will be used, with 95% confidence within an error margin of 20%, assuming a nonresponse rate of 10% of the selected facilities. If the total number of a particular type of facility for each stratum is small (<4) in the district, all facilities may be included.

For WP4, the sample size will depend on the interventions and strategies selected to be piloted and evaluated.

### Data management

Trained data entry personnel will enter the data into REDCap electronic data capture tools (https://projectredcap.org/) with regular quality checks by each site PI. For surveys conducted electronically, the data will be automatically recorded in a structured format within REDCap. After the data entry process, a thorough data cleaning procedure will be conducted to identify and correct errors, inconsistencies and missing values. Data cleaning will be achieved with a range of techniques, such as validating responses against predefined criteria, checking for outliers and resolving any discrepancies or inconsistencies. Data cleaning procedures will be documented to ensure transparency and reproducibility of the data management process. Regular backups of the data will be performed to prevent data loss and ensure data integrity. For qualitative methods, the informants’ consent, the interviews will be audio recorded, transcribed and reviewed by a researcher to ensure validity. The data will be anonymized, and identification codes will be used for confidentiality. Identifiable data will be housed under the custodianship of local PIs. All other data for purposes of health systems and economic analysis will be under the custodianship of Co-PIs in compliance with local data management policy(s), data security and sharing policy. Data managed using REDCap will be hosted and stored at IARC and will be shared with KCL through a data transfer agreement. Anonymised data will be archived in IARC to allow retrieval for any scientific or regulatory external audits.

### Data analysis

For qualitative methods, data will be analysed through the content analysis technique. This technique consists of the following three stages; pre-analysis, coding and categorisation and inference and interpretation.

The pre-analysis consists of organising the data in the district within a framework and deciding the level of analysis (word, phrase and sentence). Next, the interviews will be fully transcribed and translated to create the body of research. The coding will be done using the codification tools, and codes will be grouped into themes and categories. The team will discuss themes and types to check their reliability, and a conceptual model will be developed. Finally, we will proceed with the inference and interpretation of the results. The outcomes will be summarised to prepare a detailed report, which will help us perform the strength, weakness, opportunity and threat (SWOT) analysis and draft the capacity development response.

For quantitative methods, data cleaning and consistency checks will be performed. The facility survey data will be analysed using descriptive statistics to describe the overall capacity of health systems. For each facility and dimension, a summary score will be obtained and presented as percentages.

These reports will be compiled together for critical analysis of the strengths, weaknesses, opportunities for improvements and possible threats, particularly in augmenting the services to introduce or scale up new interventions (e.g., the introduction of HPV test or a navigation system). For the intervention phase, structural and process indicators analysed quantitatively to be used to assess the interventions objectively.

For the ISAT, the information will be synthesized (based on the data collected from the interventions, other WPs as well as from local practice-based information). The responses will generate scores that will be put in an SWOT matrix, which will allow us to understand the scalability of different interventions. The sustainability and scalability of interventions will be listed for each study site as well as visual representation of scores across each domain.

### Study monitoring

We will use a set of comprehensive input measures, milestones and outcome measures and develop and implement a dedicated information system for each WP. Site visits by the study monitor from the IARC will be arranged at study initiation and once every 6 months. Good clinical practices and Good Laboratory Practices will be implemented. Standardised questionnaires and standard operating procedures (SOP) will be developed for each of the procedures to collect data and adherence to it will be monitored regularly. Data entry and management SOPs will also be used.

### Ethical consideration

The protocol was registered prospectively on 27/9/2022 with the Clinical Trial Registry India (CTRI number CTRI/2022/09/045927). We have obtained ethical approval from the IARC Ethical Committee (IARC IEC 22-05) and will report progress for further approvals on interventions in a timely manner.

Each study site has also obtained site-specific ethical approval (HMSC 2022-17547). We will obtain written informed consent from all respondents before we begin data collection. Participation in the study is entirely voluntary. Before the intervention phase, we will obtain a separate ethical approval from each site for the final set of interventions and implementation strategies decided.

## Discussion

This study will investigate the potential to implement evidence-based interventions tailored to the specific context of three Indian states, each with distinct organisation of health system and health outcomes. Recognizing the importance of stakeholder engagement and local capacity, we aim to co-develop intervention elements and implementation strategies that are ‘best fit’ from the perspectives of the organization and populations/patients identified.

Specifically, this research will explore the feasibility of implementing effective early detection and management strategies for three distinct states with varied levels of cancer care organization maturity, providing an opportunity to assess the effectiveness of different approaches under various contexts. In Tamil Nadu, the Tamil Nadu Health Systems Project was an initiative of the government, in partnership with the World Bank, to create a health system that is highly accessible, equitable and effective. This initiative included a cervical cancer screening and treatment pilot program with good coverage. However, the programme resulted in relatively low VIA positivity, high loss to follow-up and low levels of treatment provision, likely due to gaps within the health system [37]. The pre-intervention phase of the current study will dive deep into these health system gaps and explore ways to facilitate patient follow-up and a quality-assured programme. In Kerala, the state government is currently establishing the Kerala Cancer Care Grid as part of the Kerala Cancer Control Strategy 2018–30 to ensure equitable access to affordable cancer detection and treatment services [38]. The findings of this study will support and complement this ongoing effort by the state government, recognising that any strategy proposed for the scale-up must be owned to be sustained.

The proposed effectiveness–implementation hybrid design is valuable in implementation research, enabling simultaneous evaluation of effectiveness of the proposed intervention though primarily focusing on feasibility, acceptability, adoption and maintenance of its implementation in real-world settings. This design is a pragmatic way of conducting implementation research considering the constraints policymakers and programme managers face in relation to evidence informed decision-making. It may help alleviate any public relations and political concerns that the local stakeholders might have when conducting public policy experiments by promoting collaboration, respecting local contexts, providing actionable insights and promoting sustainability.

The expected outcome of this study is to develop and evaluate a context-appropriate, evidence-based strategy that improves access to early detection and care of oral, breast and cervical cancers in rural India. The findings will provide valuable insights into how tailored interventions can reduce delays in diagnosis, improve treatment adherence and enhance overall cancer care in rural settings. If successful, these strategies can be adapted and scaled to other states across India, where similar barriers to cancer care exist. The findings from our study can serve as a model for other states with comparable challenges, enabling policymakers and health authorities to design localized, scalable cancer care interventions. This could ultimately contribute to nationwide improvements in cancer care access and outcomes, particularly in underserved rural areas, helping to bridge the urban–rural gap in healthcare delivery.

The National Programme for Prevention and Control of Cardiovascular Disease, Diabetes, Cancer and Stroke (NPCDCS) and the Ayushman Bharat program are two examples of India’s health system reforms that are intended to increase access to healthcare, notably for noncommunicable diseases such as cancer. While NPCDCS encourages cancer prevention and early detection, Ayushman Bharat’s Health and Wellness Centers concentrate on primary healthcare and cancer screening. Nevertheless, both programs encounter obstacles such as inadequate rural healthcare facilities, limited workforce and low awareness. By creating context-specific strategies to increase access to cancer screening and subsequent care in rural India, this project seeks to close these gaps and strengthen the implementation of these national initiatives [39, 40].

Several large-scale initiatives from low- and middle-income countries (LMICs) have shed light on effective implementation strategies for cancer screening and subsequent care. Recently, the WHO has laid out a strategic agenda for the effective scale-up of HPV-based screening across LMICs, including India [41]. Furthermore, recent studies have documented that successful scale-up of cancer early detection services across LMICs requires consideration of evidence-based resource stratified intervention that would fit local health infrastructure [42, 43]. The WHO Cervical Cancer Elimination Initiative also highlights the importance of establishing comprehensive health systems and community engagement to raise awareness and improve screening uptake [44]. Furthermore, strengthening partnerships among healthcare providers, local organizations and community members can enhance the effectiveness of cervical cancer prevention and treatment efforts. WHO’s recent navigation guide highlights the significance of guiding patients through early detection and treatment processes, emphasizing the need for research to identify local barriers. Identifying, implementing and integrating these elements will enhance the study’s relevance and increase its potential impact on patient outcomes and healthcare accessibility in resource-constrained settings.

Most evidence-based interventions for cancer early detection are designed, implemented and evaluated in high-income countries. These interventions and strategies are not readily transferable to LMICs. Implementation research such as this can contribute significantly to the development of cancer policies and operational frameworks by identifying context-specific challenges and developing effective strategies to overcome these challenges. In LMICs, this is particularly important, as these countries operate with limited resources and healthcare infrastructure, which can pose significant challenges to implementing effective cancer early detection programmes. With the sharing of this protocol, we aim to contribute to the evidence based on implementation science-based research related to cancer early detection, diagnosis and treatment beyond India to other similar LMIC settings.

## Conflicts of interests

The authors declare no conflicts of interest.

## Author contributions

AC, IK, KO, RI, MML, PS, RS, RR, NV, BV, MK, AP, RSN, JS, RS and PB were involved in the conception or subsequent design of the study. AC drafted the initial manuscript with inputs from IK, KO and RI. MML, AP, RSN, RS and PB provided critical inputs to the draft. All authors approved the submitted version and agree to take personal accountability for their contributions and to ensure that questions related to the accuracy or integrity of any part of the work, even ones in which the author was not personally involved, are appropriately investigated, resolved and the resolution documented in the literature.

## Ethics approval and consent to participate

We have obtained ethical approval from the IARC Ethical Committee (IARC IEC 22-05) and Health Ministry Screening Committee (HMSC, India) approval (2022-17547, approved on 30/11/2022)

## Disclaimer

Where authors are identified as personnel of the IARC/World Health Organization, the authors alone are responsible for the views expressed in this article and they do not necessarily represent the decisions, policy or views of the IARC/World Health Organization.

## ACCI Consortium

C*ancer Institute WIA, Tamil Nadu, India*

Nandimandalam Venkata Rani

Rajaraman Swaminathan *Department of Medical Oncology, GBH Group of Hospitals, Udaipur, India*

Ashish Bona

Hardika Parekh

Rohit Rebello *International Agency for Research on Cancer, Lyon, France*

Arunah Chandran

Partha Basu

Sathishrajaa Palaniraja *Karkinos Healthcare, India*

Asiya Ansari

Devu Prakash

Kunal Oswal

Moni Kuriakose

Ramadas Kunnambath

Rengaswamy Sankaranayanan

Rita Isaac *King’s College London, United Kingdom, London, UK*

Arnie Purushotham

Richard Sullivan *RTI International*

Ishu Kataria

Brian Hutchinson

Prince Bhandari

Rachel Nugent *Tata Memorial Centre, Mumbai, India*

CS Pramesh

## Supplementary Table

**Supplementary file 1.** Activities timeline.

Tasks	Year 1	Year 2	Year 3	Year 4
**WP1: Creating and engaging a stakeholder’s team**	**X**			
1.1 Formation of the stakeholder’s teams (independent at each site)	X			
1.2 Yearly meetings of the research team	X	X	X	
**WP2: Contextual factors assessment**	**X**			
2.1 Finalization of protocol and data collection tools	X			
2.2 Approval from ethics committees	X			
2.3 Completion of surveys, FGDs and KIIs	X	X		
2.4 Analysis of data and report finalization	X	X		
**WP3: Assessment of capacity of the local health systems**		**X**		
3.1 Finalizing the capacity assessment plan	X			
3.2 Data collection for landscape survey completed	X			
3.3 Facility visits completed	X	X		
3.4 Capacity development response finalized	X	X		
**WP4: Designing and costing a multilevel intervention strategy**		**X**		
4.1 Submission of the WP2 and WP3 outcomes to the stakeholder’s team meeting	X			
4.2 Finalization of protocol for pilot study		X	X	
4.3 Finalization of the logic model for evaluation of pilot		X	X	
4.4 Finalization of tools, databases etc. for the pilot		X	X	
4.5 Approval of ethics committees		X	X	
**WP5: Pilot implementation and evaluation of the intervention strategies**			**X**	
5.1 Initiation of baseline data capturing in intervention and control blocks		X		
5.2 Initiation of pilot interventions in intervention block			X	
5.3 Completion of data capturing of control block			X	X
5.4 Completion of the pilot interventions			X	X
5.5 Completion of data analysis of control and pilot			X	X
5.6 Finalization of the costing tool				X
**WP6: Assessment of the setting’s readiness and capacity to sustain and scale up the interventions**				**X**
6.1 Adaptation of the scalability assessment tools				X
6.2 Protocol finalized for scalability assessment				X
6.3 Completion of scalability assessment				X
**Final analysis of data and publication of results**				X
